# Osteoblastic lesion screening with an advanced post-processing package enabling in-plane rib reading in CT-images

**DOI:** 10.1186/s12880-016-0141-0

**Published:** 2016-05-20

**Authors:** Hannes Seuss, Peter Dankerl, Alexander Cavallaro, Michael Uder, Matthias Hammon

**Affiliations:** Department of Radiology, University Hospital Erlangen, Friedrich-Alexander-Universität (FAU) Erlangen-Nürnberg, Maximiliansplatz 1, 91054 Erlangen, Germany

**Keywords:** Computed tomography, Bone neoplasm, Workflow, Image processing, In-plane, Rib, Osteoblastic

## Abstract

**Background:**

To evaluate screening and diagnostic accuracy for the detection of osteoblastic rib lesions using an advanced post-processing package enabling in-plane rib reading in CT-images.

**Methods:**

We retrospectively assessed the CT-data of 60 consecutive prostate cancer patients by applying dedicated software enabling in-plane rib reading. Reading the conventional multiplanar reconstructions was considered to be the reference standard. To simulate clinical practice, the reader was given 10 s to screen for sclerotic rib lesions in each patient applying both approaches. Afterwards, every rib was evaluated individually with both approaches without a time limit. Sensitivities, specificities, positive/negative predictive values and the time needed for detection were calculated depending on the lesion’s size (largest diameter < 5 mm, 5–10 mm, > 10 mm).

**Results:**

In 53 of 60 patients, all ribs were properly displayed in plane, in five patients ribs were partially displayed correctly, and in two patients none of the ribs were displayed correctly. During the 10-s screening approach all patients with sclerotic rib lesions were correctly identified reading the in-plane images (including the patients without a correct rib segmentation), whereas 14 of 23 patients were correctly identified reading conventional multiplanar images. Overall screening sensitivity, specificity, and positive/negative predictive values were 100/27.0/46.0/100 %, respectively, for in-plane reading and 60.9/100/100/80.4 %, respectively, for multiplanar reading. Overall diagnostic (no time limit) sensitivity, specificity, and positive/negative predictive values of in-plane reading were 97.8/92.8/74.6/99.5 %, respectively. False positive results predominantly occurred for lesions <5 mm in size.

**Conclusions:**

In-plane reading of the ribs allows reliable detection of osteoblastic lesions for screening purposes. The limited specificity results from false positives predominantly occurring for small lesions.

## Background

The most common type of malignant bone tumors found are metastases. These originate from various cancer types with high incidence and prevalence, such as prostate cancer, breast cancer and colon cancer [1]. Up to 70 % of prostate cancer patients develop bone metastases, which are typically osteoblastic/sclerotic in nature [2, 3]. Most patients do not die because of the growth of the primary cancer, but rather because of its spread to other sites [4]. Bone metastases portend a poor survival, with a median of less than 6 months [5] and, therefore, are of critical importance for oncological patients. Accurate detection of bone metastases is thus an important task in a radiologist’s daily routine, because it provides valuable clinical information, which enables the timely choice of systemic or local therapies, such as surgical intervention or radiation. There are different manifestations of bone metastases; they can appear osteolytic or osteoblastic/sclerotic in nature or show a mixed appearance [6]. The presence of bone metastases can provide an important prognostic factor of whether the patient will benefit from chemotherapy, which is often associated with impairing side effects [7].

Nowadays, there are many radiological methods available for examination of the skeletal system for osseous metastases, such as computed tomography (CT), magnetic resonance imaging (MRI), bone scintigraphy, single-photon emission CT (SPECT) or positron emission tomography CT (PET-CT) [8]. However, in routine clinical practice, the initial staging, and especially the follow-up examinations of oncological patients, often include CT imaging only. CT demonstrates superior bone detail, allowing early detection of bone metastases [9]. Nevertheless, it is challenging and time-consuming to detect bone lesions at an early stage on CT-images, especially in the ribs because of their curved shapes. Moreover, it has been postulated that skeletal metastases are at risk of being missed because bone windows are underutilized in a radiologist’s daily routine [10]. Hence, a dedicated software package supporting the reader in detecting osteoblastic rib lesions in CT-images can be regarded as a needed and useful tool in the diagnosis, staging and treatment of cancer patients. It could also assist the reader in making final decisions. It can be part of an often demanded multipurpose computer-aided detection system [11]. To efficiently utilize the software in daily practice, it is crucial to achieve a reliable display of the lesions in one plane, which enables a more user-friendly mode for quick reporting.

Therefore, this study was conducted to evaluate the screening and diagnostic accuracy and efficiency for the detection of osteoblastic rib lesions in CT-images using an advanced post-processing package enabling in-plane rib reading compared to a conventional multiplanar reading approach.

## Methods

This retrospective study was conducted in accordance with the guidelines of the Declaration of Helsinki and approved by the Ethics Committee of the University Hospital Erlangen. The need for written informed consent was waived by the Ethics Committee.

### Patient population

For this retrospective study, the hospital information system (HIS) was used to search for the last 60 patients with histologically confirmed prostate cancer who underwent thoraco-abdomino-pelvic CT imaging. We retrospectively evaluated 60 consecutive patients who matched these parameters (male; mean age, 72 years; range, 48–89 years). In the analyzed period, four patients were scanned twice and one patient three times. Only the first examination was used for the study. Twenty-three of the 60 patients showed osteoblastic rib lesions; 37 patients showed no rib lesions. A secondary cancer was not known to be present in any of the patients.

### Imaging technique

CT examinations were performed with a Somatom Sensation 64-detector row system (Siemens AG, Erlangen, Germany) with the following parameters: craniocaudal thoraco-abdomino-pelvic CT-data acquisition, 120 kV, Care Dose (Siemens, Erlangen, Germany); pitch, 0.9; collimation, 0.6 mm; section thickness, 1 mm; and bone reconstruction kernel. Images were acquired in portal venous phase (intravenous application of weight-adapted, warmed Iomeron 400 (Bracco Imaging, Konstanz, Germany) followed by a saline flush with a flow rate of 3 ml/s through a 18- or 20-gauge catheter in an antecubital vein. Participants were imaged in the supine position.

### Experimental setup

**Fig. 1:**
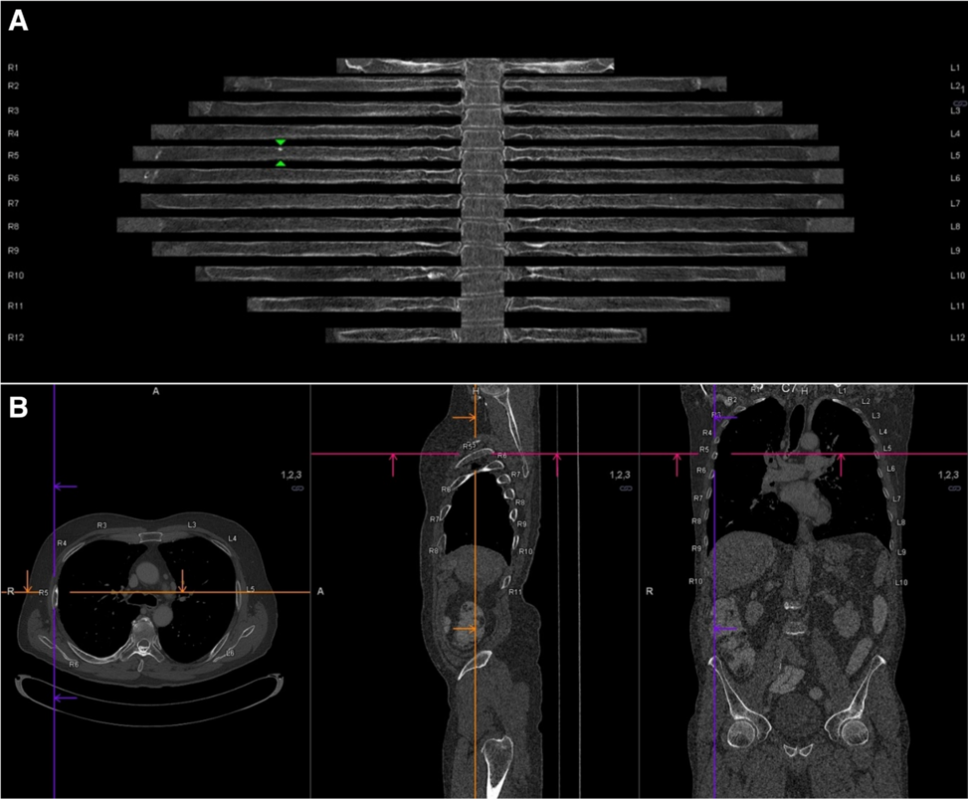
**a** Example of a post-processed in-plane image of the ribs. A small sclerotic lesion is visible in the 5^th^ right rib (*green arrows*). **b** Multiplanar display of the corresponding thoraco-abdomino-pelvic CT-scan (*bone window*) in axial, sagittal and coronal planes, with a small sclerotic lesion in the 5^th^ right rib (localized with hairline crosses)

Quantitative image-data analysis was performed using dedicated, commercially available software enabling in-plane rib reading in CT-data (syngo.via, Siemens AG, Erlangen, Germany). If automated segmentation was performed correctly, a spider-like image was generated with the vertebral column as the body and the 24 ribs as perpendicular extremities (Fig. 1a). By scrolling through the image, the reader can swivel the ribs while the vertebrae remain fixed. The vertebrae and ribs of each side are labeled with numbers from 1 to 12. The labels are constantly displayed in every plane and reformation next to the ribs. All images possess spatial information and can be collocated by clicking into the image or by using a slider to navigate along the axis of one rib. By default, the software generates 36 consecutive in-plane images of the entire ribcage with a 10° rotation along the curved planar reformatted main axis of each rib. Presets can be changed in steps of one degree if different angles are desired.

An osteoblastic/sclerotic lesion was defined as a spherical, hyperdense lesion within the cancellous bone or deriving from the compact bone bulging inward. Tubular structures were considered trabeculae or united fractures and were not considered. Osteolytic lesions were also not considered. A rib was considered to be successfully segmented and displayed when there was no discontinuity or uncharacteristic presentation.

An experienced reader with 5 years of work experience retrieved and analyzed the CT-data. A research assistant prepared the blinded studies in random order and recorded the findings and times. The reader was blinded to the patients’ characteristics and to clinical information. Failed in-plane presentation or labeling of the ribs was documented. First, the reader evaluated each study for 10 s (to simulate a screening approach) and subsequently had to decide if at least one sclerotic rib lesion was present (per-patient analysis). In a second step, every rib was read separately without time limitations and each sclerotic lesion was recorded and marked (per-rib analysis). Additionally, the evaluation time was documented. These evaluations were performed for both approaches (conventional reading using multiplanar reconstructions (Fig. 1b) and reading the post-processed in-plane images). Evaluations were performed 30 to 41 days apart to avoid recall bias. Finally, we analyzed if reading one in-plane image of the longest axis or reading two in-plane images of the longest and shortest axes were sufficient for screening purposes. Since no measurements can be performed using the in-plane images, measurements of all marked lesions were performed in the multiplanar images after the evaluation process. The lesions were measured in three dimensions and grouped according to their largest diameters (largest diameter <5 mm = small lesion, largest diameter of 5–10 mm = medium lesion, largest diameter >10 mm = large lesion).

### Statistical analysis

Statistical analysis was performed using dedicated software (SPSS Statistics v20, IBM Corp., Armonk, NY, USA). Sensitivities, specificities, and positive and negative predictive values with corresponding confidence intervals were calculated for the detection of osteoblastic rib lesions using an advanced post-processing package enabling in-plane rib reading in CT-images (screening and diagnostic accuracy were calculated separately). Additionally, the screening accuracy was determined for the conventional multiplanar reading approach. Diagnostic multiplanar reading with a dedicated workstation and without time limit was considered to be the reference standard. False positive and false negative results that occurred during in-plane image evaluation were analyzed. Patient characteristics are expressed as means ± standard deviations along with ranges.

## Results

### Standard of reference (multiplanar read)

**Fig. 2:**
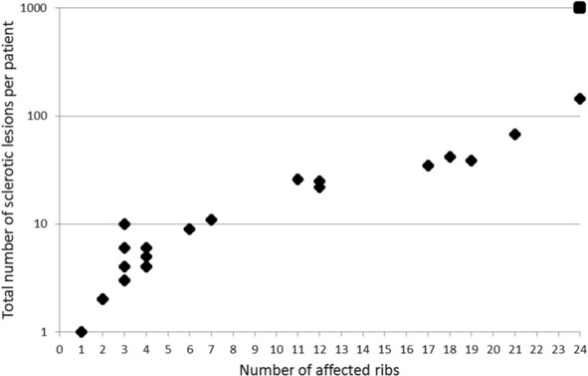
Number of affected ribs. Scatter plot of a per-patient analysis of sclerotic lesions. The x-axis represents the number of ribs that show at least one sclerotic lesion and the y-axis represents the total number of lesions in all affected ribs in each patient (*n* = 21). Patients with diffuse sclerotic lesions (*n = 2)* are represented by the square (the number of sclerotic lesions was not countable and therefore set to 1000). Patients without sclerotic lesions (*n* = 37) are not displayed

A total of 473 sclerotic lesions were found in 183 ribs in 21 patients. In an additional two patients, diffuse, unmeasureable osteoblastic lesions were present in all ribs (Fig. 2). Therefore, 23 of 60 patients presented with at least one sclerotic rib lesion (median of 9.5 lesions per patient; range: 1 lesion per patient to diffuse sclerosis). On average, 9.6 ribs had at least one sclerosis with a median of five ribs (range of 1 to 24). A total of 116 lesions were classified as large (largest diameter >10 mm), 134 as medium (largest diameter = 5–10 mm) and 223 as small (largest diameter <5 mm).

### Rib-segmentation quality (in-plane images)

**Fig. 3:**
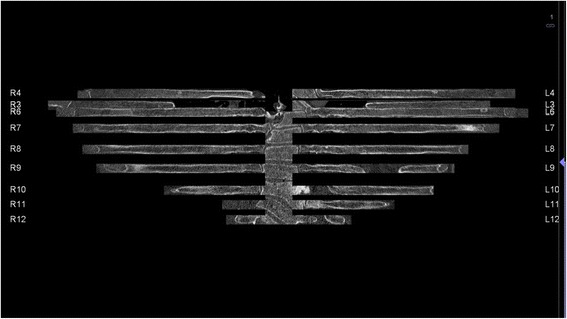
Examples of rib segmentation errors, e.g. oblique display of the 11^th^ or 12^th^ vertebra, incomplete display of ribs with holes (e.g. 9^th^ *left rib*), and display of ribs in the wrong order (4^th^, 3^rd^, 6^th^ ribs on both sides)

**Fig. 4:**
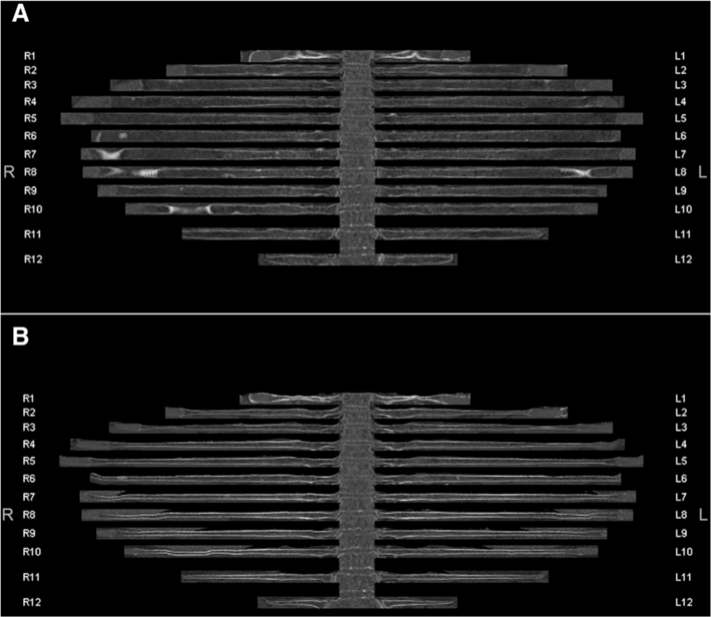
In-plane image of the entire rib cage displaying the long (**a**) and the short (**b**) axis of each rib. The sclerotic lesion in the 6^th^ rib on the right can clearly be seen in both axes. There are regions with artifacts in the long axis image (**a**) due to old fractures causing slight kinking of the rib contours (R1, R7, R8, R10, L1 and L8). These regions can be better analyzed in the short axis image (**b**)

The software successfully displayed the complete rib cage in plane in 53 of 60 patients (88 %) and 1349 of 1440 ribs (94 %). In five patients, 77 of 120 ribs were displayed correctly in plane and in two patients none of the ribs were displayed correctly in plane, resulting in a total of 91 failed rib segmentations in 60 patients. Most errors occurred in the segmentation of the first rib with a total of 12 false segmentations (10 %). Detailed information is given in Table 1. Examples of failed rib segmentations are shown in Fig. 3. The value of long and short axis is demonstrated in Fig. 4.

**Table 1:** In-plane rib segmentation quality based on a per-rib and per-patient analysis

	Number of correctly segmented ribs	
Rib (*n* = 1440)	Right	Left
*First*	54/60 (90 %)	54/60 (90 %)
*Second*	56/60 (93 %)	56/60 (93 %)
*Third*	57/60 (95 %)	56/60 (93 %)
*Fourth*	57/60 (95 %)	57/60 (95 %)
*Fifth*	57/60 (95 %)	56/60 (93 %)
*Sixth*	57/60 (95 %)	57/60 (95 %)
*Seventh*	57/60 (95 %)	56/60 (93 %)
*Eighth*	57/60 (95 %)	57/60 (95 %)
*Ninth*	57/60 (95 %)	56/60 (93 %)
*Tenth*	57/60 (95 %)	56/60 (93 %)
*Eleventh*	56/60 (93 %)	56/60 (93 %)
*Twelfth*	55/60 (92 %)	55/60 (92 %)
*Total*	677/720 (94.0 %)	672/720 (93.3 %)
Patient (*n* = 60)		
*Number 3*	8/24	
*Number 6*	3/24	
*Number 13*	22/24	
*Number 14*	22/24	
*Number 26*	22/24	
*Number 36*	0/24	
*Number 51*	0/24	
*Remaining patients*	1272/1272

### Ten-second screening

**Fig. 5:**
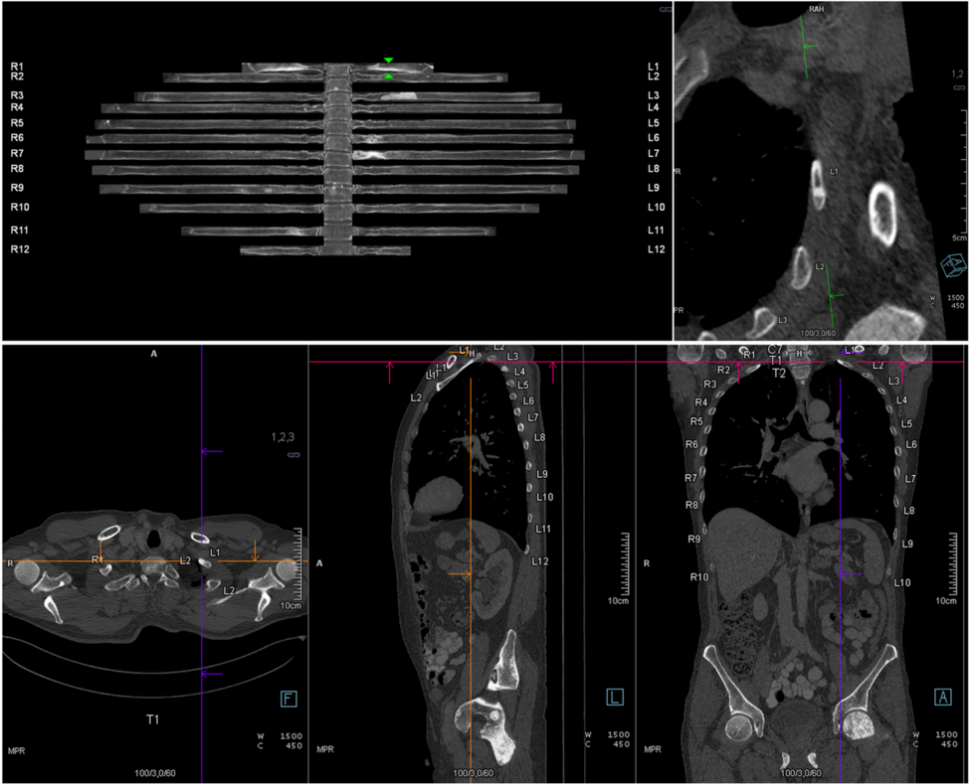
Example of a false negative result in in-plane reading. The 1^st^ *left rib* is partially cropped. The sclerotic lesion is visible in the multiplanar reconstructions but not displayed in the in-plane reconstruction

Using post-processed in-plane images only, all 23 patients with sclerotic lesions were identified within ten seconds (sensitivity: 100 %), compared to 14 identified patients using the conventional axial images (sensitivity: 60.9 %). Due to artifacts from reconstruction algorithms, 27 patients were classified as false positives during the in-plane reading (specificity: 27.0 %) compared to no false positives using the conventional reading approach. This results in a positive and negative predictive value of 46.0 % and 100 %, respectively, for in-plane reading (conventional reading: 100 % and 80.4 %, respectively). Detailed information is shown in Table 2. An example of a true positive and false negative result is shown in Figs. 1 and 5.

**Table 2:** Screening accuracy based on a per-patient analysis was determined by reading each patient’s in-plane and multiplanar images for 10 seconds and diagnostic accuracy based on a per-rib analysis was determined for reading in-plane images without time constraint

	Ten-second screening accuracy on a per-patient analysis		Diagnostic reading accuracy on a per-rib analysis (no time constraint)
	In-plane reading	Multiplanar reading	In-plane reading
*True positives*	23	14	226
*True negatives*	10	37	993
*False positives*	27	0	77
*False negatives*	0	9	5
*Sensitivity*	100 % (82.1–100 %)	60.9 % (38.8–79.5 %)	97.8 % (94.7–99.2 %)
*Specificity*	27.0 % (14.4–44.4 %)	100 % (88.3–100 %)	92.8 % (91.0–94.2 %)
*Positive predictive value*	46.0 % (32.1–60.5 %)	100 % (73.2–100 %)	74.6 % (69.2–79.3 %)
*Negative predictive value*	100 % (65.5–100 %)	80.4 % (65.6–90.1 %)	99.5 % (98.8–99.8 %)

Multiplanar image analysis of every rib without time constraint was considered to be the reference standard. Confidence intervals are shown in brackets. N = 60 patients/1440 ribs

### Diagnostic in-plane reading without time constraint

Based on a per-rib analysis, 226 of 1440 analyzed ribs were found to be true positives during in-plane reading (including the two patients with diffuse sclerotic rib lesions), 993 ribs were found to be true negatives, 77 ribs were false positives and five ribs false negatives. This results in a sensitivity of 97.8 %, a specificity of 92.8 %, a positive predictive value of 74.6 % and a negative predictive value of 99.5 % (Table 2). The reasons for false negative results obtained during diagnostic in-plane reading are described in Table 3.

**Table 3:** Reasons for the 5 false negative results during diagnostic in-plane reading

Rib	Reason for false negative results during diagnostic in-plane reading
*First*	The maximum diameter of the rib was larger than the segmented area. One lesion was not within the segmented area
*First*	The maximum diameter of the rib was larger than the segmented area. Two lesions were not within the segmented area
*Eighth*	Small lesion was mistaken for a trabecula
*Tenth*	Lesion was not within the segmented area
*Eleventh*	Small sclerotic lesion at the cartilage bone junction was overlooked

No time constraint. Per-rib analysis. The first example is depicted in Figure 5. N = 60 patients/1440 ribs

Analysis regarding lesion size did yield 128 ribs that had at least one large lesion, 120 ribs with medium lesions and 162 ribs with small lesions, resulting in a diagnostic sensitivity of 97.8 % for large, 98.4 % for medium and 97.0 % for small lesions. A total of 77 normal ribs were misdiagnosed as positive and 73 of the false positive ribs were due to small lesions.

**Fig. 6:**
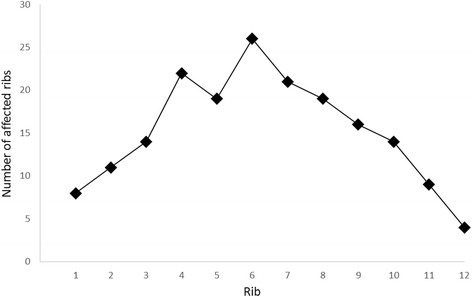
Number of affected ribs dependent on the kind of rib (1^st^ – 12^th^ rib)

Furthermore, sensitivities, specificities, and positive and negative predictive values were calculated for each type of rib (1^st^ to 12^th^ rib). While sensitivities, specificities and negative predictive values were relatively consistent, positive predictive values of the smaller ribs (especially the 1^st^ and 12^th^ rib) were significantly lower than those of the larger ribs (*p* < 0.01) (Table 4) There is a strong correlation between the size of the rib and the prevalence of sclerotic lesions (Fig. 6).

**Table 4:** True and false positives, true and false negatives, sensitivities, specificities, positive predictive values (PPV) and negative predictive values (NPV) for in-plane image reading based on a per-rib analysis

**Rib**	**True positives**	**True negatives**	**False positives**	**False negatives**	**Affected ribs**
*1*	10	84	13	2	12
*2*	15	88	6	0	15
*3*	18	87	4	0	18
*4*	26	77	6	0	26
*5*	23	78	8	0	23
*6*	30	71	7	0	30
*7*	25	78	5	0	25
*8*	22	80	5	1	23
*9*	20	83	5	0	20
*10*	17	88	2	1	18
*11*	12	85	10	1	13
*12*	8	94	6	0	8
*All*	226	993	77	5	231
	**Sensitivity**	**Specificity**	**PPV**	**NPV**	
*1*	83.3 %	86.6 %	43.5 %	97.7 %	
*2*	100 %	93.6 %	71.4 %	100 %	
*3*	100 %	95.6 %	81.8 %	100 %	
*4*	100 %	92.8 %	81.3 %	100 %	
*5*	100 %	90.7 %	74.2 %	100 %	
*6*	100 %	91.0 %	81.1 %	100 %	
*7*	100 %	94.0 %	83.3 %	100 %	
*8*	95.7 %	94.1 %	81.5 %	98.8 %	
*9*	100 %	94.3 %	80.0 %	100 %	
*10*	94.4 %	97.8 %	89.5 %	98.9 %	
*11*	92.3 %	89.5 %	54.5 %	98.8 %	
*12*	100 %	94.0 %	57.1 %	100 %	
*All*	97.8 %	92.8 %	74.6 %	99.5 %

### Single/dual in-plane image reading

The reader successfully detected 96.5 % of the true positive ribs (reference standard: in-plane read) by reading just one in-plane reformatted image (long axis). Reading an additional in-plane image (90° rotated; short axis) further improved the detection of the true positive ribs to 98.7 %, which resulted in a sensitivity of 94.4 % for the detection of positive ribs (reference standard: multiplanar read) with one in-plane image and a sensitivity of 96.5 % with two perpendicular in-plane images.

### Reading time

**Fig. 7:**
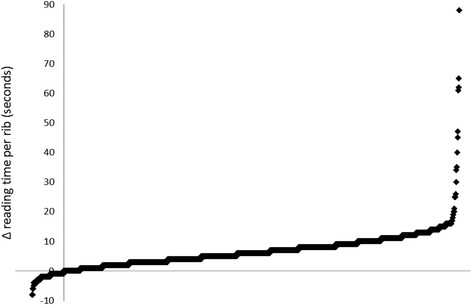
Reading time difference based on a per-rib analysis (multiplanar reading time minus in-plane reading time), arranged in ascending order. Mean reading time difference was 6.3 s per-rib. The median difference was 6 s, with the range from - 8 to 88 s. Points in the upper right area of the diagram represent faster readings with in-plane images, the points in the lower left vice versa

The mean evaluation time per rib was 13.9 ± 7.5 s during conventional multiplanar reading and 7.5 ± 4.2 s during post-processed in-plane image reading. The fastest rib analysis took 2 s with both methods, whereas the longest rib evaluation took 111 s during multiplanar reading and 61 s during in-plane reading (Fig. 7).

## Discussion

In a cohort consisting of 60 patients with prostate cancer, a total of 473 sclerotic rib lesions were found in 23 patients. The software correctly displayed 94 % of the ribs in plane. In 88 % of patients all ribs were displayed correctly in plane. As demonstrated, all patients with suspected sclerotic lesions were identified within ten seconds using the post-processed in-plane images compared to 61 % using the classical workflow. By reading just one in-plane reformatted image, 96.5 % of the ribs with sclerotic lesions were correctly identified. The average reading time for one rib was 7.5 s using the in-plane images and 13.9 s using the conventional multiplanar images.

Sclerotic lesions demask early on, but the task is to detect them while only having a limited amount of time for examining the whole torso. Many computer-aided detection (CAD) systems are commercially available or currently being developed, but they are still seldomly integrated into daily routine. We evaluated post-processing software that presents the whole rib cage in in-plane images to facilitate screening for osseous lesions or fractures. After identifying one lesion and being primed to explicitly look for rib metastases, the reader is able to focus on the bones and may find additional lesions. Such a piece of software seems to be valuable for daily practice because the bone window is known to be under-represented during the read of CT-exams [12]. Nevertheless, during cancer staging and follow-up exams, a thorough examination is vital for therapy planning and patient outcome [4, 5, 7].

We chose a collective of patients with known prostate cancer because of a relatively high prevalence of sclerotic metastases [13]. Although a sclerotic bone lesion is not necessarily a metastasis, we did not feel the necessity to further clarify the lesions with scintigraphy or SPECT/PET-CT for this study because we wanted to simulate a typical clinical routine workflow in which the reader reports suspected sclerotic osseous lesions using CT-images alone. Therefore, we were explicitly looking for CT-morphologically suspected sclerotic lesions and not for confirmed osteoblastic metastases.

In the per-patient screening examination the number of false positives was higher than the true positives (positive predictive value 46 %). This is due to the reformation of the images, which creates artifacts from, for example, bifid ribs, united fractures or simply geometric enlargements that cannot be reliably differentiated from suspected lesions. One might argue that flipping a coin yields better results; however, it is still an excellent tool for screening purposes with a sensitivity and a negative predictive value of 100 %. Furthermore, the predominant false positives resulted from small hyperdense dots (<5 mm). One approach could be to ignore any sporadic small hyperdense dots found on the images. The software enables navigation from the finding in the in-plane images to the corresponding region in the multiplanar images. The software facilitates an instant presentation of the lesion in four different layers (axial, coronal, sagittal and perpendicular to the main axis of the rib) and the artifacts can be ruled out straightforwardly, a feature that was excluded by the design of our study in order to assess the quality of the in-plane images alone.

If every rib is assessed individually, only five ribs were considered normal though presenting a lesion. In one of those cases, the reader simply overlooked the lesion and one other lesion was mistaken for a trabecula. In three ribs the false negative result was due to the segmentation process. Two first ribs were wider than the segmented area, with the lesions lying within this unsegmented area. In one tenth rib, the generated axis was not completely centered in all layers and again the lesion was excluded from the segmentation. It must be noted that more than those five lesions were missed. However, there was at least one other finding, so those ribs were still correctly diagnosed as suspect. It is understood that especially small lesions were missed, particularly if many suspected findings were present and therefore did not alter the result. With regards to the segmentation quality, the small ribs showed the worst diagnostic accuracy. In particular, the positive predictive value of the first rib was found to be 44 %, which is far below the average of 75 %. The few real findings in those small ribs were plagued with many artifacts due to the reasons described above.

In this experimental setting, the mean time to evaluate one rib was 7.5 s, adding up to a total theoretical time of three minutes for the entire rib cage. The analysis in the multiplanar mode took nearly twice as long. However, it must be noted that the mean time per-rib did vary widely, depending on the number of sclerotic lesions, particularly if overlapping lesions were present (e.g., it was difficult to evaluate if one large or two medium lesions were present). Furthermore, every single lesion had to be marked, measured and recorded. In both methods, the fastest analysis of one rib took two seconds, which was only possible with small ribs and if no findings needed to be recorded. The longest time for the analysis was 111 s. This rib and one other outlier that took 87 s to analyze were in the same patient with an extremly high amount of lesions. In a clinical setting, a time-consuming complete analysis is not neccesary, as it is only important to describe the overall impression as “one”, “few”, “many”, “diffuse” and measurement of some target lesions, a task that can be done within minutes after the initial lesion is detected. Using the reformatted in-plane images this task can be accomplished within seconds.

The reformatted image is roughly twice as wide as high. If viewed in a quadratic window the magnification is low or the image has to be adjusted by zooming in and moving it around. One possible advancement could be to divide the ribcage into left and right sections and then export the two series so that both sides can be displayed fully magnified. Other anatomical regions could be targeted as well to create new kinds of images, all based on an in-plane reformation, thereby potentially facilitating more efficient bone window reading. The skull could be flattened like a map, analogous to a globe. The pelvis is also a common site of metastases. The pelvic girdle could also be opened like a book and displayed in plane.

Failed rib segmentation can be identified easily, e.g. due to holes or interruptions in the corresponding rib, and even complete ribs can be missing. Other incorrect segmentations include, breaching of the borders of the row by the compacta or oblique segmented vertebrae. In most of the cases, an incorrect segmentation is the result of an underlying pathology, such as diffuse metastases, osteoporosis, extreme kyphosis, closeness to other bones like the scapula and clavicula, or the presence of a foreign body (e.g. a pacemaker). Though not present in the patient population the algorithm was also tested with different anatomical anomalies. Lumbar ribs were not recognized by the software as ribs. Fork ribs were detected, however only one path was reconstructed in the final image. An osseous spur between two ribs was ignored and cut from the final image. The examinations were performed in the portal venous phase with potential problems due to beam hardening artifacts. However, we attribute no failed rib segmentation to beam hardening artifacts in the evaluated cohort.

Special attention must be dedicated to the first rib. Ten percent of the first ribs were not segmented correctly. Reasons for this reduced performance might be the high degree of curvature, low circularity and the large maximum diameter of the first rib. Two of the five false negative results were located in the first ribs. The large diameter of these ribs breached the area of the reformatted image and in two cases the sclerotic lesions were not displayed/visible. Segmentation of the smaller ribs seems to be more challenging. Staal et al. omitted these ribs in their segmentation process [14]. Another influencing factor in the segmentation process of the upper ribs is the closely located scapula [15].

There is a strong correlation between the size of the rib and the prevalence of sclerotic lesions. Figure 6 shows the number of affected ribs by the kind of rib (1^st^ – 12^th^ rib).

Our study faces several limitations that suggest possible directions for future work. First, we restricted our cohort to prostate cancer patients only. This collective was chosen because of the high incidence of sclerotic osseous lesions that enabled us to lower the number of patients and still get a decent amount of findings to evaluate. We specifically described the findings as sclerotic lesions and not as metastases to simulate clinical practice CT reading. To make a more general statement about the software performance it could be evaluated with other cohorts or with a large collective without any preselection. Another constraint of this study was the limitation to sclerotic lesions. During the evaluation we noticed that in-plane images might also be very appropriate to detect osteolytic osseous lesions, which may be metastases of cancers, such as multiple myeloma or renal cell carcinoma. Moreover, additional studies focusing on inter-reader variability need to be conducted to evaluate reproducibility.

## Conclusions

Automatically post-processed in-plane images of the ribs enable efficient screening for sclerotic lesions in CT data, which is important during the evaluation of every CT exam, including the thorax, and especially during the staging of cancer patients. The limited specificity resulted from false positives predominantly occurring for small lesions (largest diameter <5 mm). In-plane images can be automatically transferred to the Picture Archiving and Communication System and therefore be seamlessly integrated into the clinical workflow, which potentially facilitates a more efficient bone window reading.

## Abbreviations

CT: computed tomography
MRI: magnetic resonance imaging
SPECT: single-photon emission computed tomography
PET-CT: positron emission tomography computed tomography
HIS: hospital information system
CAD: computer-aided detection
